# Supplementary material for: Perioperative Complications of Anterior Decompression with Fusion in Degenerative Cervical Myelopathy—A Comparative Study between Ossification of Posterior Longitudinal Ligament and Cervical Spondylotic Myelopathy Using a Nationwide Inpatient Database
Source: J Clin Med. 2022 Jun 13;11(12):3398. doi: 10.3390/jcm11123398 (PMC9225569; doi:10.3390/jcm11123398)
Supplement: Supplementary file 1 [file jcm-11-03398-s001.zip › Supplementary Table S1.pdf]

Supplementary Table S1. Systemic complications and local complications between ≥ 70 and < 70 in OPLL after matching.

**Systemic complications**	**≥ 70 (N=295)**	**< 70 (N=902)**	***P* value**
At least one systemic complication	58 (19.7%)	90 (10.0%)	<0.001***
Cardiovascular disease	6 (2.0%)	26 (2.9%)	0.43
Cerebrovascular disease	3 (1.0%)	6 (0.7%)	0.54
Respiratory failure	6 (2.0%)	7 (0.8%)	0.07
Pneumonia	7 (2.4%)	4 (0.4%)	0.003**
Dysphagia	12 (4.1%)	14 (1.6%)	0.010*
Renal failure	1 (0.3%)	2 (0.2%)	0.73
Hepatic failure	1 (0.3%)	1 (0.1%)	0.41
Deep venous thrombosis	1 (0.3%)	2 (0.2%)	0.73
Pulmonary embolism	1 (0.3%)	0 (0%)	0.08
Sepsis	2 (0.7%)	0 (0%)	0.013*
Delirium	3 (1.0%)	0 (0%)	0.002**
**Local complications**	**≥ 70 (N=295)**	**< 70 (N=902)**	***P* value**
At least one local complication	15 (5.1%)	58 (6.4%)	0.40
Surgical site infection	5 (1.7%)	20 (2.2%)	0.59
Paralysis	3 (1.0%)	11 (1.2%)	0.78
Meningitis	0 (0%)	2 (0.2%)	0.42
Spinal fluid leakage	6 (2.0%)	26 (2.9%)	0.43
Hematoma	1 (0.3%)	6 (0.7%)	0.52

Data were presented as n (%). Significant values are given as follows. $^{*}P<0.05$, $^{**}P<0.01$, $^{***}P<0.001$

OPLL, ossification of posterior longitudinal ligament.
