# Supplementary material for: Perioperative Complications of Anterior Decompression with Fusion in Degenerative Cervical Myelopathy—A Comparative Study between Ossification of Posterior Longitudinal Ligament and Cervical Spondylotic Myelopathy Using a Nationwide Inpatient Database
Source: J Clin Med. 2022 Jun 13;11(12):3398. doi: 10.3390/jcm11123398 (PMC9225569; doi:10.3390/jcm11123398)
Supplement: Supplementary file 1 [file jcm-11-03398-s001.zip › Supplementary Table S2.pdf]

Supplementary Table S2. Systemic complications and local complications between ≥ 70 and < 70 in CSM after matching.

**Systemic complications**	**≥ 70 (N=356)**	**< 70 (N=841)**	***P* value**
At least one systemic complication	62 (17.4%)	84 (10.0%)	<0.001***
Cardiovascular disease	21 (5.9%)	14 (1.7%)	<0.001***
Cerebrovascular disease	3 (0.8%)	6 (0.7%)	0.81
Respiratory failure	7 (2.0%)	9 (1.1%)	0.22
Pneumonia	8 (2.3%)	5 (0.6%)	0.012*
Dysphagia	11 (3.1%)	14 (1.7%)	0.12
Renal failure	0 (0%)	0 (0%)	NA
Hepatic failure	1 (0.3%)	5 (0.6%)	0.48
Deep venous thrombosis	2 (0.6%)	5 (0.6%)	0.95
Pulmonary embolism	0 (0%)	0 (0%)	NA
Sepsis	3 (0.8%)	3 (0.4%)	0.28
Delirium	2 (0.6%)	1 (0.1%)	0.16
**Local complications**	**≥ 70 (N=356)**	**< 70 (N=841)**	***P* value**
At least one local complication	11 (3.1%)	24 (2.9%)	0.83
Surgical site infection	3 (0.8%)	8 (1.0%)	0.86
Paralysis	2 (0.6%)	7 (0.8%)	0.62
Meningitis	0 (0%)	0 (0%)	NA
Spinal fluid leakage	1 (0.3%)	3 (0.4%)	0.84
Hematoma	5 (1.4%)	7 (0.8%)	0.36

Data were presented as n (%). Significant values are given as follows. *P<0.05, ***P<0.001

CSM, cervical spondylotic myelopathy; NA, not applicable.
