# Supplementary material for: Perioperative Complications of Anterior Decompression with Fusion in Degenerative Cervical Myelopathy—A Comparative Study between Ossification of Posterior Longitudinal Ligament and Cervical Spondylotic Myelopathy Using a Nationwide Inpatient Database
Source: J Clin Med. 2022 Jun 13;11(12):3398. doi: 10.3390/jcm11123398 (PMC9225569; doi:10.3390/jcm11123398)
Supplement: Supplementary file 1 [file jcm-11-03398-s001.zip › Supplementary Table S3.pdf]

Supplementary Table 3. Cost and length of stay without perioperative complications between OPLL and CSM after matching.

**Total complication (-)**	**OPLL (N=988)**	**CSM (N=1,020)**	***P* value**
Cost ($)	18,058 ± 7,959	15,492 ± 6,791	<0.001***
Length of stay (days)	26.8 ± 19.6	22.9 ± 18.8	<0.001***
**Systemic complication (-)**	**OPLL (N=1,049)**	**CSM (N=1,051)**	***P* value**
Cost ($)	18,289 ± 8,229	15,584 ± 6,839	<0.001***
Length of stay (days)	27.3 ± 20.1	23.1 ± 18.8	<0.001***
**Local complication (-)**	**OPLL (N=1,124)**	**CSM (N=1,162)**	***P* value**
Cost ($)	18,626 ± 8,904	16,188 ± 8,670	<0.001***
Length of stay (days)	28.1 ± 21.7	25.2 ± 32.1	0.012*

Data were presented as mean ± SD. Significant values are given as follows. *P<0.05, ***P<0.001

OPLL, ossification of posterior longitudinal ligament; CSM, cervical spondylotic myelopathy; SD, standard deviation.
